# Minimum material requirements for hand hygiene in community settings: a systematic review

**DOI:** 10.1136/bmjgh-2025-018926

**Published:** 2025-09-16

**Authors:** Lilly A O’Brien, Kennedy Files, Jedidiah S Snyder, Hannah K Rogers, Oliver Cumming, Joanna Esteves Mills, Bruce Gordon, Matthew C Freeman, Bethany A Caruso, Marlene K Wolfe

**Affiliations:** 1 Gangarosa Department of Environmental Health, Rollins School of Public Health, Emory University, Atlanta, Georgia, USA; 2 Woodruff Health Sciences Center Library, Emory University, Atlanta, Georgia, USA; 3 Department of Disease Control, London School of Hygiene & Tropical Medicine, London, UK; 4 Water, Sanitation, Hygiene and Health Unit, World Health Organization, Geneva, Switzerland; 5 Hubert Department of Global Health, Rollins School of Public Health, Emory University, Atlanta, Georgia, USA

**Keywords:** Hygiene, Public Health, Prevention strategies

## Abstract

**Background:**

This systematic review assessed the minimum requirements necessary to create an enabling environment for sustained hand hygiene practices: quantity of water and soap, and number, spacing, location, and design of hand hygiene facilities.

**Methods:**

We searched PubMed, Web of Science, EMBASE, CINAHL, Global Health, Cochrane Library, Global Index Medicus, Scopus, PAIS Index, WHO IRIS, UN Digital Library and World Bank eLibrary, and consulted experts. Eligible studies were published through 29 March 2023, observational, in non-healthcare community settings, and reported on at least one of the five categories: (1) quantity of water, (2) quantity of soap, (3) location of hand hygiene materials, (4) number of users or spacing of facilities and (5) considerations for equitable access. Two reviewers independently extracted data from each study and assessed risk of bias using the Mixed Method Appraisal Tool.

**Results:**

This review identified 37 studies that met inclusion criteria from 27 countries, representing 4 of the 6 WHO regions (Africa, South-East Asia, the Americas and Europe). Household settings were the most represented (59% of studies), followed by institutional or school settings (41%) and public establishments (27%). Of the 37 studies, 12 (32%) assessed the relationship between a material requirement and hand hygiene practices. Despite extensive global research on hand hygiene, we found a lack of evidence linking material requirements with handwashing practices in community settings.

**Conclusions:**

This review was limited to observational studies, and more data could be derived from experimental studies. Important evidence gaps include the quantity of water and soap needed, the influence of facility location and design on hand hygiene practice, and material needs providing equitable access. Further research is needed to strengthen the evidence base for hand hygiene recommendations and supplement the expert opinion on which many recommendations are currently based.

**PROSPERO registration number:**

CRD42023429145.

**WHAT IS ALREADY KNOWN ON THIS TOPIC**

- Hand hygiene is an important tool to prevent infectious disease transmission; however, there is little information about what the minimum material requirements are to enable handwashing in community settings to achieve this goal.

**WHAT THIS STUDY ADDS**

- This systematic review examines the evidence of material needs to enable handwashing in community settings and the relationship with handwashing-associated outcomes when reported in included studies. It demonstrates that there is very little evidence providing practical, yet critical, information on what materials are needed to meet handwashing needs in community settings.
- This review highlights key gaps in the current evidence base, particularly surrounding the specifics of quantities and locations of materials. Gathering information on these gaps is a necessity for increasing our understanding and providing recommendations on the minimum requirements for effective and sustained hand hygiene in community settings.

**HOW THIS STUDY MIGHT AFFECT RESEARCH, PRACTICE OR POLICY**

- This review guides future hand hygiene studies to key questions to intentionally collect data on, based on the identified gaps, and urges those studies to examine the association between these material needs and hand hygiene practice.
- More detailed and specific reporting of the materials and conditions associated with handwashing, alongside evidence about practice and health and other outcomes, is needed to improve recommendations.
- Beyond reporting more quantitative details, there was a lack of studies focused on equitable access to handwashing, and this review calls for future research to specifically address material needs for handwashing for people with disabilities.

## Introduction

 Hand hygiene, which includes handwashing with soap and other methods such as the use of alcohol-based hand rubs (ABHRs), plays a critical role in preventing many infectious diseases including diarrhoeal and respiratory infections, which account for a large burden of disease.^1^,^3^ In addition to impacting individual health and quality of life, these infections place a resource strain on healthcare systems and result in substantial costs, especially in low-income and middle-income countries where preventable infectious diseases remain a leading cause of morbidity and mortality.^4^,^6^ Despite the importance of hand hygiene and unprecedented levels of attention and investment towards infrastructure and behaviour change during the COVID-19 pandemic,^7^ there continues to be insufficient access to basic services for hand hygiene, especially in low-resource settings.^8^,^10^ Hand hygiene interventions are relatively inexpensive to implement, suggesting a high potential for cost-effectiveness in mitigating infectious disease transmission and resulting effects.^11^ Establishing global guidelines and recommendations is essential to guide hand hygiene initiatives, protect public health and strengthen health systems.^12^

Despite the well-documented effectiveness and benefits of hand hygiene,^713^,^15^ a recent scoping review identified key gaps and inconsistencies in international guidelines on hand hygiene regarding the minimum material requirements needed to support sustained hand hygiene practices, efficacy of different materials, behaviour change approaches and government measures.^16^ For minimum material requirements, gaps were identified in recommendations on the quantity of water and soap needed, locations and spacing of hand hygiene facilities, and considerations for equitable access to these requirements.^16^ While there is broad agreement on the efficacy of soap and water and ABHRs for hand hygiene,^16 17^ hand hygiene can take many forms depending on the availability and usage of different materials, and the specific requirements of these variations are often not enumerated in recommendations.

Hand hygiene facility design can vary widely—examples include running water from a tap, a tippy-tap, a water pitcher or basin, paired with bar soap, soapy water, liquid soap, powder soap, a stand with a bottle of ABHR and more. The quantity of water used while handwashing varies with the type of facility due to factors such as how easily the flow and flow rate is controlled by the user, the design’s efficiency in conserving water, and the user’s perception of water availability through the facility.^18^ It is widely acknowledged that the amount of water used for domestic purposes is substantially influenced by accessibility, continuity, reliability and price of water.^19^

Maintaining effective hand hygiene requires not only presence of necessary materials at a single moment of observation but their consistent availability over time. Facilities and materials used for hand hygiene often serve additional purposes, such as for drinking, cooking and cleaning. These competing priorities may impede adoption of recommended hand hygiene practices, particularly when there is limited access to needed materials. This is especially pertinent for domestic settings where use of soap and water for hand hygiene is deprioritised in favour of other essential activities. Studies in Egypt, Ethiopia, Kenya and Uganda found that these materials were prioritised for laundry, cooking, bathing and dishes over handwashing, with some participants noting that their hands were being cleaned while completing these other tasks.^20^,^23^ This dynamic is further supported through a companion review by our team focused on barriers and enablers to hand hygiene where we identified ‘water prioritisation’ for other activities and ‘water supply quantity’ as barriers to handwashing, specifically in domestic settings.^24^ Though recent publications and reports have noted insufficient empirical evidence to define minimum quantities of water necessary for basic needs including hygiene, expert opinion on the quantity varies from 15 to 20 L/person/day.^19 25^ However, these estimates do not take into account what materials are available for handwashing or what other practices may be priorities in the community.^26^ Competing demands for resources used in hand hygiene underscore the need for evidence-based guidelines on minimum material requirements that not only make hand hygiene physically possible but sustainably enable the practice in real-world settings.^16^

The aim of this systematic review was to assess the minimum requirements reported in observational studies of hand hygiene for sustained practice of effective hand hygiene in community settings. The priority question and subquestions for this review were generated through an extensive consultation process by the WHO with external experts,^27 28^ following the aforementioned scoping review of current international guidelines.^16^

## Methods

### Research questions

This systematic review sought to assess the following five questions related to minimum requirements for hand hygiene (online supplemental S1 – RQ in SPIDER Format): (a) What quantity of water is required to enable handwashing with soap and water at key moments? (b) What quantity of soap is required to enable handwashing with soap and water at key moments? (c) Where should soap and water or alternatives be located in community settings to enable hand hygiene at key moments? (d) What is the optimal spacing and number of users per hand hygiene facility in household settings and public places to enable hand hygiene with soap and water at key moments? (e) What are the main considerations for ensuring equitable access to minimum material requirements and preventing discrimination in community settings?

### Search strategy

This review was preregistered with PROSPERO (registration number: CRD42023429145) and is reported in accordance with the Preferred Reporting Items for Systematic Reviews and Meta-Analyses (PRISMA) criteria (online supplemental S2 – PRISMA Checklist).^29^ This review was part of an integrated protocol for multiple related reviews to synthesise the evidence for effective hand hygiene in community settings.^28^ We adopted a two-phased approach for identifying relevant studies. Phase 1 involved a broad search to capture all studies on hand hygiene in community settings that were relevant across multiple related systematic reviews. The outcome of phase 1 was a reduced sample from which further screening specific to this review, referred to as phase 2, was performed. A full description of the procedures followed for searches, study inclusion, outcomes data collection, analysis and reporting of the multiple related reviews is presented in the published protocol.^28^

The phase 1 database search included studies published through 29 March 2023 that were published in English or had titles and abstracts published in English. We searched 12 peer-reviewed and grey literature databases. PubMed, Web of Science, EMBASE (Elsevier), CINAHL (EBSCOhost), Global Health (CAB), Cochrane Library, Global Index Medicus, Scopus (Elsevier), Public Affairs Information Service (PAIS) Index (ProQuest) were searched on 23 March 2023 and WHO Institutional Repository for Information Sharing (IRIS), UN Digital Library and World Bank eLibrary were searched on 28 March 2023 using search terms related to hand hygiene broadly and restrictions on terms related to healthcare settings in the titles. We searched trial registries (International Clinical Trials Registry Platform and ClinicalTrials.gov) for trials related to hand hygiene in community settings on 29 March 2023.

In addition, we contacted 35 content experts and organisations, using snowballing methods, from April to May 2023 for information on relevant unpublished literature. Manual searches of reference lists were not completed for this review as we did not identify existing systematic reviews relevant to the research question.

### Selection criteria

For this review, hand hygiene refers to any hand cleansing undertaken for the purpose of removing or deactivating pathogens from hands and efficacious hand hygiene is defined as any practice which effectively removes or deactivates pathogens from hands and thereby has the potential to limit disease transmission.^30^ The term community settings included domestic (eg, households), public (eg, markets, public transportation hubs, vulnerable populations (eg, people experiencing homelessness), parks, squares, or other public outdoor spaces, shops, restaurants, and cafes), and institutional (eg, workplace, schools and universities, places of worship, prisons and places of detention, nursing homes and long-term care facilities) spaces.^16^ Studies were excluded if they were in healthcare settings or were animal research. Studies focusing on care providers’ hand hygiene in nursing homes, long-term care facilities and home-based care settings were excluded as part of phase 2 screening as the evidence they generated was determined to be similar to that from healthcare settings. Non-English language studies were excluded during screening due to the research team’s limited proficiency to understand other languages and accurately interpret the data presented. There were no geographical restrictions.

Quantitative and mixed-methods studies were eligible for inclusion. Publications not based on empirical research were excluded. Studies were included if the topic of research was on the quantity of water and/or soap required for handwashing with soap at key moments both as recommended and as commonly practised; the location of soap and water or alternatives to enable hand hygiene at key moments; the spacing and number of users per hand hygiene facility required for handwashing with soap and water at key moments; or considerations (including location and design) leading to harm or inequitable access to handwashing with soap at key moments or discrimination.

We used Covidence software for systematic reviews.^31^ In both phases, screening of each article (phase 1—title and abstract only; phase 2—title and abstract, then full text review) was performed independently by two reviewers, with discordance between reviewers reconciled by a third reviewer. The stages and related outcomes of the search and screening process are described in the PRISMA flow diagram (online supplemental S3 – PRISMA Flow Diagram).

### Data analysis

Two reviewers (LAO’B and KF) independently extracted data using customised data extraction tools (onlinesupplemental S4  Covidence extraction and S5 )^32^ and assessed quality for each article using the Mixed Method Appraisal Tool (MMAT).^33 34^ Any conflicts between reviewers over data extraction and bias assessment were resolved by discussion and checking the source material.

We extracted qualitative data describing the location and design of handwashing facilities along with quantitative data on the amounts of water and soap reported for use and the number of users and spacing per hand hygiene facility. We also extracted quantitative data on the statistical association between these observations and measures of hand hygiene practice. These findings were summarised narratively and in tables, when sufficient data were reported. We established location categories based on observed data without predetermined classifications due to uncertainty about the locations that would be represented in the data. We intended to conduct quantitative summaries and meta-analyses for each subquestion in this review. However, we were unable to do so in a meaningful way due to the limited amount of data and/or the extent of the variation in how each material requirement of interest and the outcome of hand hygiene were measured and reported across studies.

### Patient and public involvement

Patients or the public were not involved directly in the design, or conduct, or reporting, or dissemination plans of our research. This evidence synthesis supports the forthcoming WHO Guidelines for Hand Hygiene in Community Settings; the study questions were developed in broad consultation with a network of key partners. Findings from this review will be disseminated alongside the guidelines.

## Results

### Study characteristics of the studies included in this review

**Figure 1:**
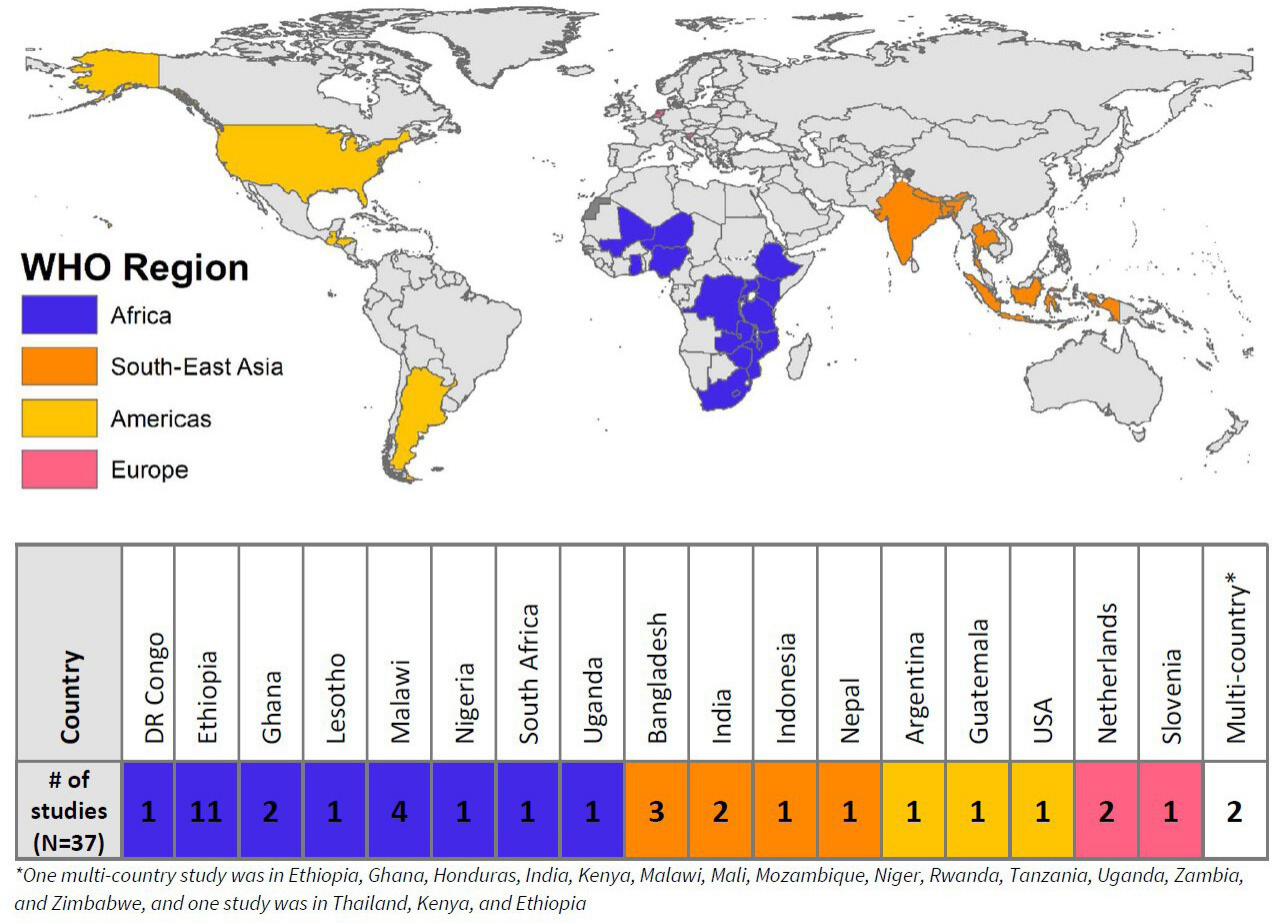
Global distribution of studies reporting on minimum material requirements for hand hygiene in community settings. Indonesia is shown in the South-East Asia Region to maintain consistency with our analysis period; WHO reclassified Indonesia to the Western Pacific Region in May 2025.

We identified 37 unique studies that met inclusion criteria (table 1). Studies took place in 27 countries, representing 4 of the 6 WHO regions—Africa (n=24 studies), South-East Asia (n=9), the Americas (n=4) and Europe (n=3) (figure 1 and table 2). We did not identify any studies from the Eastern Mediterranean region or Western Pacific Region. Domestic, or household, settings were the most represented (in 59% of studies), followed by institutional settings (41%) and public settings (27%). The most common primary outcome was the practice of hand hygiene (62%), followed by different health outcomes (16%). Among institutional settings, primary and secondary schools were most commonly studied, followed by daycare centres and universities. The most frequently described hand hygiene practice was handwashing with soap and water (54%). Table 1 summarises the characteristics of the studies in greater detail.

**Table 1:** Summary of the included studies

a. Quantity of water available (no identified studies)							
b. Quantity of soap available							
Study ID	Community Setting	Setting type	Country	Urban/rural setting	Quantity of soap distributed, as presented in the study	Handwashing impact assessed?	MMAT Score
Biran *et al*^35^	Refugee Camp	Household	Kenya	n/a	No soap distribution	No	5
			Ethiopia	n/a	1 bar/person/month		
			Thailand	n/a	4 bars/person/3 months		
Peterson *et al*^36^	Refugee Camp	Household	Malawi	n/a	240 g/person/month	No	4

c. Location of hand hygiene facilities							
Study ID	Community Setting	Setting type	Country	Urban/rural setting	Locations assessed, as presented in the study	Handwashing impact assessed?	MMAT Score
Adane *et al*^37^	Domestic	Households	Ethiopia	Urban	Within or near latrines	No	3
Admasie and Debebe^38^	Domestic	Households	Ethiopia	Urban	In or around the latrine	No	3
Admasie *et al*^39^	Domestic and Institutional	Households and Schools	Ethiopia	Urban and Rural	At the home (household); In the school compound (school)	Yes	4
Agaro *et al*^40^	Domestic	Households	Ethiopia	Rural	Near to latrine; Near to household; not a separate place	No	5
Aithal *et al*^41^	Domestic	Households	India	Urban and Rural	Toilet; bathroom; outside	Yes	4
Belachew *et al*^42^	Domestic	Households	Ethiopia	Rural	Near to the toilet	No	4
Berhanu *et al*^43^	Domestic and Institutional	Households and Schools	Ethiopia	Urban and Rural	Next to latrines (household); presence of a hand washing facility in a student’s home (household); In compound within walking distance (school); Within walking distance (school)	Yes	4
Besha *et al*^44^	Institutional	Schools	Ethiopia	Urban and Rural	Outside latrine room	No	4
Chidziwisano *et al*^45^	Domestic	Households	Malawi	Rural	Near the latrine; In the household	Yes	5
Fielmua *et al*^46^	Public	Markets and Shops	Ghana	Urban	Handwashing stations around the entrance; Hand sanitiser stations around the entrance	No	5
Green *et al*^47^	Public	Restaurants	USA	Not reported	In worker’s sight	Yes	5
Jubayer *et al*^48^	Domestic	Households	Bangladesh	Not reported	On household premises	No	5
Kalam *et al*^49^	Domestic	Households	Bangladesh	Urban	Within 10 ft of a latrine or toilet	Yes	5
Kalumbi *et al*^50^	Domestic	Households	Malawi	Not reported	A fixed handwashing facility within the plot/yard; mobile facility (basin and jug used in multiple locations)	No	5
Luby *et al*^51^	Domestic	Households	Bangladesh	Rural	Inside or near the toilet facility; inside or near the kitchen; elsewhere in the yard; No specific place; outside the yard	Yes	5
Melaku and Addis^52^	Institutional	Schools	Ethiopia	Not reported	Toilets; lounges and dining areas; school yard	No	5
Moffa *et al*^53^	Domestic	Households	See footnotes for countries*	Rural	Within 5 m of the food preparation area; Within 5 m of the latrine; neither location	No	5
Mugambe *et al* ^54^	Public	Supermarkets	Uganda	Urban	Entrance of supermarket	No	5
Mwapasa *et al* ^55^	Institutional	Childhood development centres	Malawi	Peri-urban	At the food preparation area; near the latrines	No	5
Natnael *et al* ^56^	Public	Barbershops and beauty salons	Ethiopia	Not reported	Presence of hand-washing facility with water and soap; presence of hand-washing facility that is convenient and user friendly in/near the barbershop/the beauty salon	Yes	4
Prevolšek *et al*^57^	Public	Food vendor stalls	Slovenia	Urban	Suitably located washbasins	No	5
Rao *et al*^58^	Domestic	Households	Guatemala	Not reported	Within 10 m of sanitation facility	No	5
Ray *et al*^59^	Domestic and Institutional	Households and schools	India	Urban	Near the toilets (household); near the toilet (school)	No	5
Serra and Soria ^60^	Institutional	Daycare centres	Argentina	Urban	In the day care centre	No	5
Shrestha *et a*^61^	Domestic	Households	Nepal	Not reported	Inside toilet; inside kitchen; within courtyard; other	No	4
Sibiya and Gumbo^62^	Institutional	Schools	South Africa	Urban and Rural	Inside their toilets; at the centre of schools, about 100 m from the toilet	No	4
Sondari *et al*^63^	Institutional	Schools	Indonesia	Not reported	Facilities present	Yes	3
Tessema and Alemu^64^	Domestic	Households	Ethiopia	Not reported	Hand washing facility available	Yes	3
van Beeck *et al*^65^	Institutional	Daycare centres	Netherlands	Urban and Rural	All facilities were present in playroom (soap, sink and towel)	No	5
Wami *et al* ^66^	Institutional	Schools	Nigeria	Urban	At the entrance of the school	No	4
Zomer *et al*^67^	Institutional	Daycare centres	Netherlands	Urban and Rural	At least one sink in each classroom	No	5

d. Number of facilities per area or user							
Study ID	Community Setting	Setting type	Country	Urban/rural setting	Number of facilities per area or user, as presented in the study	Handwashing impact assessed?	MMAT Score
George *et al*^68^	Public and Institutional	15 indoor public location types	Democratic Republic of the Congo	Urban	Multiple hand sinks	No	4
Green *et al*^47^	Public	Restaurants	USA	Not reported	Multiple handwashing stations	Yes	5
van Beeck *et al*^65^	Institutional	Daycare centres	Netherlands	Urban and Rural	2 sinks with taps per playroom	No	5
Zomer *et al*^67^	Institutional	Daycare centres	Netherlands	Urban and Rural	2 sinks, towel facilities, and soap facilities per classroom per 2 caregivers	Yes	5

e. Design considerations							
Study ID	Community Setting	Setting type	Country	Urban/rural setting	Design of facility, as presented in the study	Handwashing impact assessed?	MMAT Score
Kalam *et al*^49^	Domestic	Households	Bangladesh	Urban	Presence of a permanent handwashing facility in the home	Yes	5
Kebede *et al*^69^	Domestic	Households	Ethiopia	Urban and Rural	Observed, fixed place; observed, mobile place	Yes	5
Letuka *et al*^70^	Public	Markets	Lesotho	Urban	Buckets to pour water over hands	No	5
Sondari *et al*^63^	Institutional	Schools	Indonesia	Not reported	Hand washing facilities with permanent buildings	No	3
Steiner-Asiedu *et al*^71^	Domestic and Institutional	Households and Schools	Ghana	Urban	Sinks with running water (households and schools); receptors for communal handwashing (households and schools); plastic containers with a tap (households and schools)	No	4
Wami *et al*^66^	Institutional	Schools	Nigeria	Urban	Standpipe	No	4
Zomer *et al*^67^	Institutional	Daycare centres	Netherlands	Urban and Rural	Paper towels or fabric towels; soap pump or bar soap or soap dispenser	Yes	4

* Ethiopia, Ghana, Honduras, India, Kenya, Malawi, Mali, Mozambique, Niger, Rwanda, Tanzania, Uganda, Zambia, and Zimbabwe

MMAT, Mixed Method Appraisal Tool; n/a, not available.

**Table 2:** Characteristics of the included studies

Descriptive characteristics of studies*	Total n (%)	Quantitative n (%)	Mixed methods n (%)
Total number of studies	37 (100)	25 (65)	12 (35)
Region			
African Region	24 (65)	14 (56)	10 (83)
South-East Asian Region	9 (24)	7 (28)	2 (17)
Region of the Americas	4 (11)	3 (12)	1 (8)
European Region	3 (8)	3 (12)	–
Eastern Mediterranean Region	–	–	–
Western Pacific Region	–	–	–
Multiple regions	2 (5)	1 (4)	1 (8)
Urban/rural			
Urban	12 (32)	7 (28)	5 (42)
Rural	5 (14)	4 (16)	1 (8)
Periurban	1 (3)	–	1 (8)
Other: refugee camp	2 (5)	1 (4)	1 (8)
Both urban and rural	8 (22)	7 (28)	1 (8)
Unspecified	9 (24)	6 (24)	3 (25)
Setting			
Domestic (households)	22 (59)	16 (64)	6 (50)
Institutional	15 (41)	10 (40)	5 (42)
Daycare centre	4 (11)	3 (12)	1 (8)
Primary and secondary schools	10 (27)	6 (24)	4 (33)
Universities	1 (3)	1 (4)	–
Public	10 (27)	5 (20)	5 (42)
Restaurant	2 (5)	1 (4)	1 (8)
Food vendor stalls	1 (3)	–	1 (8)
Barbershops and beauty salons	2 (5)	–	2 (17)
Markets	4 (11)	4 (16)	-
Places of worship	1 (3)	1 (4)	–
Sex of primary research population			
Female	3 (8)	2 (8)	1 (8)
Male	–	–	–
Unspecified or both male and female	34 (92)	23 (92)	11 (92)
Primary outcome studied			
Hand hygiene	23 (62)	14 (56)	9 (75)
Health outcome	6 (16)	6 (24)	–
Diarrhoeal diseases	4 (11)	4 (16)	–
Nutrition	1 (3)	1 (4)	–
Neglected tropical diseases	1 (3)	1 (4)	–
WASH access or KAP	5 (14)	1 (4)	4 (33)
COVID-19 KAP	3 (8)	3 (12)	–
Food hygiene	2 (5)	2 (8)	-
Hand hygiene practice			
Handwashing with soap and water	20 (54)	10 (40)	10 (83)
Handwashing with water	4 (11)	2 (8)	2 (17)
Handwashing with soap and water or water only	4 (11)	3 (12)	1 (8)
Hand washing with soap and water or ash	2 (5)	2 (8)	–
Handwashing with soap and water or ABHR	2 (5)	2 (8)	–
Handwashing with ABHR	1 (3)	–	1 (8)
More than 2 methods	5 (14)	4 (16)	1 (8)
Handwashing—unspecified	2 (5)	2 (8)	–
Study quality rating (mean)	4.46	4.40	4.58

* Descriptive statistics for region, setting, primary outcome studied and hand hygiene practice represent variables that were multiselect, so the sum of sections may be over the study total.

ABHRs, alcohol-based hand rubs; KAP, Knowledge, attitude, and practice; WASH, water, sanitation, hygiene.

### Quality of the studies included in this review

The mean study quality was 4.46 overall, indicating good quality, 4.40 for quantitative descriptive or non-randomised studies (n=25), and 4.58 for mixed-methods studies (n=12). All studies received a score of 3 or higher. Online supplemental S6 – MMAT Assessment presents quality appraisal scores for each study included.

#### Quantity of water

We did not identify any observational studies that reported on the quantity of water available or required to enable hand hygiene at key moments in community settings.

#### Quantity of soap

No studies reported the quantity of soap available or required to enable handwashing in community settings, but two studies (table 1) reported the quantity of soap distributed in refugee settings.^35 36^ We excluded a priori studies that noted only presence or absence of soap, as our research questions sought to determine quantity rather than presence. Peterson *et al* reported that 240 g of soap were distributed per person per month at a refugee camp in Malawi,^36^ and Biran *et al* reported that no soap distribution occurred at a camp in Kenya, one bar of soap per person was distributed at a camp in Ethiopia, and four bars of soap per person were distributed every 3 months at a camp in Thailand.^35^ Biran *et al* also reported on the prevalence of both handwashing with water only and handwashing with soap by refugee camp, but neither study carried out an analysis of the association between the quantity of soap distributed and hand hygiene behaviour. The observed percentage of potential handwashing events that occurred with both water and soap was low overall, but was highest in Ethiopia (19%), followed by Kenya (17%) and then Thailand (15%).^35^

#### Location of hand hygiene facilities

31 studies reported on the observed or self-reported location of hand hygiene facilities in domestic settings (households), institutional settings (schools and daycare centres) or public settings (markets, supermarkets, restaurants, food vendors, and barber and beauty shops) (table 1).^37^,^67^ 10 individual studies reported the associations between location of hand hygiene facilities and hand hygiene practices (table 3 and online supplemental S7 - Facility location associations).

**Table 3:** Association of locations of hand hygiene facilities with hand hygiene practices

Location	Studies n (%)*	Association with hand hygiene practices							
		Positive†	MMAT	Negative†	MMAT	No association identified†	MMAT	Not assessed†	MMAT
Domestic settings	17	Average MMAT: 4.29							
In or around a latrine or toilet	13 (76)	Aithal *et al* ^41^ Kalam *et al* ^49^	4 5	–	–	Luby *et al*^51^‡	5	Adane *et al*^37^ Admasie and Debebe^38^ Agaro *et al*^40^ Belachew *et al*^42^ Berhanu *et al*^43^ Chidziwisano *et al* ^45^ Moffa *et al*^53^ Rao *et al*^58^ Ray *et al*^59^ Shrestha *et al*^61^	3 3 5 4 4 5 5 5 5 4
On household premises	6 (35)	–	–	Aithal *et al*^41^	4	Luby *et al*^51^‡	5	Agaro *et al*^40^ Jubayer *et al*^48^ Kalumbi *et al*^50^ Shrestha *et al*^61^	5 5 5 4
Present but unspecified location	6 (35)	Chidziwisano *et al*^45^	5	–	–	Luby *et al*^51^‡ Tessema and Alemu ^64^	5 3	Agaro *et al*^40^ Moffa *et al*^53^ Shrestha *et al*^61^	5 5 4
In a kitchen or food preparation area	3 (18)	–	–	–	–	Luby *et al*^51^‡	5	Moffa *et al*^53^ Shrestha *et al*^61^	5 4
In the home	3 (18)	Berhanu *et al*^43^	4					Admasie *et al*^39^ Chidziwisano *et al*^45^	4 5
Outside household premises	1 (6)	–	–	–	–	Luby *et al*^51^‡	5	–	–
Institutional settings	12	Average MMAT: 4.42							
On school premises	6 (50)	Admasie *et al*^39^ Sondari *et al*^63^	4 3	–	–	–	–	Berhanu *et al*^43^ Melaku and Addis^52^ Sibiya and Gumbo^62^	4 5 4
In or around a latrine or toilet	5 (42)	–	–	–	–	–	–	Besha *et al*^44^ Melaku and Addis^52^ Mwapasa *et al*^55^ Ray *et al*^59^ Sibiya and Gumbo^62^	4 5 5 5 4
In a kitchen or food preparation area	2 (17)	–	–	–	–	–	–	Melaku and Addis^52^ Mwapasa *et al* ^55^	5 5
In the school	2 (17)	–	–	–	–	–	–	Serra and Soria ^60^ van Beeck *et al*^65^ Zomer *et al*^67^	5 5 5
Outside school premises	1 (8)	–	–	–	–	–	–	Berhanu *et al*^43^	4
At entrance of school	1 (8)	–	–	–	–	–	–	Wami *et al*^66^	4
Public settings	5	Average MMAT: 4.80							
Nearby to workers	3 (60)	Green *et al*^47^	5	–	–	Natnael *et al* 2022^56^	4	Prevolšek *et al*^57^	5
At the entrance	2 (40)	–	–	–	–	–	–	Fielmua *et al*^46^ Mugambe *et al*^54^	5 5
On-premises	1 (20)	Natnael *et al*^56^	4	–	–	–	–	–	–

Studies are categorised by whether they found a positive, negative or no association between presence of facilities in a location and the performance of hand hygiene practices, as defined and reported by the individual studies.

* The percentage represents the portion of studies in a specific setting (domestic, institutional or public) that look at a particular location grouping.

† Unless otherwise stated, the reference category for the association was absence of a handwashing station in the assessed location.

‡ ‘Outside the yard’ is the reference group for all Luby 2009 associations.

MMAT, Mixed Method Appraisal Tool.

Hand hygiene facility locations were categorised into the following categories for domestic settings (categories were based on extracted data): in or around a latrine or toilet; in a kitchen or food preparation area; in the home (not specified to be in/around a latrine/toilet or in a kitchen area); on household premises (either in the yard or near the home); outside household premises, but available to participants; unspecified location, but available to participants. Locations of hand hygiene facilities in institutional settings were categorised as: in or around a latrine or toilet; in a kitchen or food preparation area; in a school (not specified to be in/around a latrine/toilet or in a kitchen area); on school premises (either noted to be in the school yard or present without a specified location); outside school premises, but available to students; or at the entrance of the school. Resulting locations reported from public settings were categorised as: nearby to workers (in sight or easily accessible); at the entrance of a public establishment; or on-premises of a public establishment.

Of the studies in domestic settings, 76% recorded whether a handwashing station was observed ‘in or around a latrine or toilet’ (table 3). The next most common location assessed was the presence of a hand hygiene facility ‘on household premises’ or the facility was ‘present but (in an) unspecified location’ (each was 35% of studies). Two studies found positive associations between the location of ‘in or around a latrine or toilet’ and hand hygiene practices compared with no facility at the location. Aithal *et al* found that a higher proportion of mothers practised hand hygiene when the wash area was in or near the toilet compared with outside the toilet.^41^ Similarly, Kalam *et al* found a higher proportion practised hand hygiene when there was a handwashing facility within 10 feet of a toilet compared with the absence of a toilet in that location.^49^ Chidziwisano *et al* found that caregivers who had a handwashing facility present reported to wash hands with soap more frequently than those without a facility.^45^ Berhanu *et al* also found positive associations between reported hand hygiene practices and having a facility in a student’s home.^43^ Luby *et al* reported null associations between hand hygiene practices for the location ‘inside or near the toilet’ and other locations, specifically when ‘outside the yard’ was the reference group.^51^ Tessema and Alemu reported null associations between ‘hygiene practices’ and the presence or absence of a facility, noting that their measure of ‘hygiene practices’ was an aggregate measure that also included non-hand hygiene measures (online supplemental S7 – Facility location association).^64^

In the institutional settings, 50% of studies considered the simple presence or absence of a facility, and 42% considered a location of in or around a latrine or toilet (table 3). Two studies found a positive association between having hand washing facilities on school premises and improved hand hygiene practices when compared with having no facilities available; improved hand hygiene was defined based on two different composite scoring systems that both considered performance of handwashing at critical times, using appropriate materials and frequency (online supplemental S7 – Facility location association).^39 63^

In public settings, one study (Green 2007, food vendors) found a positive association between having a facility nearby to a worker and ‘appropriate’ hand washing practices (as defined by the paper, including removal of gloves, handwashing with soap and water, and drying) being used,^47^ but another (Natnael 2022, barber shops and beauty salons during COVID-19 pandemic) reported a null association for the same location.^56^ Conversely, Natnael 2022 found a positive association between increased hand hygiene practices and the presence of a hand hygiene facility compared with no facility present (online supplemental S7 – Facility location association).^56^

#### Number of facilities per area or users

Four studies reported on factors related to the number of facilities per area or per users, and two studies reported on associations of these factors with hand hygiene practices (table 1).^47 65 67 68^ Green *et al* reported that food workers were more likely to ‘appropriately’ wash their hands (definition above) when multiple hand sinks were available in a restaurant compared with one or fewer sinks.^47^ Zomer *et al* reported that an increased number of towel facilities per caregiver was positively associated with hand hygiene compliance, but the number of sink facilities and soap facilities per caregiver was not associated with hygiene outcomes (online supplemental S8 – Number of facilities associations).^67^

#### Design considerations of hand hygiene facilities

Seven studies reported on the design of the hand hygiene facilities specifically, and three studies reported on associations of these factors with hand hygiene practices (table 1).^4963 66 67 69^,^71^ Both Kalam *et al* and Kebede *et al* found that the odds of users practising hand hygiene were higher when the facility was a fixed or permanent design compared with a mobile facility.^49 69^ Zomer *et al* reported that caregivers in a daycare centre were more likely to wash their hands when there were only paper towels available compared with either fabric towels or both paper towels and fabric towels. They did not find an association between types of soap facilities available (soap pump, soap bar or soap dispenser) and handwashing behaviours (online supplemental S9 – Design associations).^67^

## Discussion

Our review of the minimum requirements necessary to enable sustained, effective handwashing in community settings found a lack of evidence to describe the material requirements in terms of quantity of soap and water, location, spacing and design of handwashing stations—and even less evidence relating these material needs to hand hygiene practices. Although the literature was too sparse for consensus to emerge from the available data, one area in which our findings were consistent was that the presence of any hand hygiene facility in community settings supports sustained handwashing. Overall, the lack of sufficient evidence across the literature regarding these minimum requirements and their association with hand hygiene is a critical gap, as this information is important in informing future recommendations and interventions to improve hand hygiene. This review can serve as a foundation for the development of key research questions and considerations within interventional studies focusing on hand hygiene practices.

### Quantity of water

We found no data that specified the quantity of water available and used for handwashing in the studies included in this review, and therefore, we found no data reporting on the association between observed quantities of water and hand hygiene practices. The quantity of water available to an individual may influence the frequency and quality of handwashing practice. The volume of water used for handwashing also varies depending on the type of hand hygiene station used, such as a running water tap which typically uses more water than a basin. These materials may, in turn, be related to other factors associated with water scarcity. Studies have shown that hand hygiene interventions are only effective at reducing mortality in non-water scarce settings and when there is a predictable water supply available.^72 73^ This epidemiological evidence supports the critical role of water in hand hygiene and emphasises the importance of understanding water supply and scarcity beyond presence and absence.

While during the screening process, we found many studies that reported on the presence or absence of water available for handwashing, these studies did not provide any information on water volume. Presence or absence information may provide insight into overall access to water; however, it does not capture scarcity nor enable quantification of the amount of water needed to enable effective handwashing as defined by WHO guidelines. Although recommendations for water quantity requirements exist based on expert opinion and estimations, these are few and discordant.^16^ Future studies should aim to quantify the amount of water available and used for handwashing, even if roughly estimated, or use proxy measures to help determine water supply needs for handwashing.

As collecting data on the volume of water present at a location may be difficult or infeasible in many settings, we suggest that future researchers consider using alternative or proxy variables that can give insights and be generalised to provide recommendations. These could be measures looking at water source, reliability, scarcity and other related variables, noting the selection of measures should be informed by contextual factors relevant to the research setting.

In domestic settings, standardised and globally validated tools such as the Household Water InSecurity Experiences (HWISE) Scale or Individual Water InSecurity Experiences (IWISE) Scale could be used.^74 75^ Because these scales represent water security as a high-level aggregate score, they allow for data across different contexts to be compared, and recommendations can be developed on minimum thresholds. By recommending minimum thresholds on comprehensive scores from tools such as HWISE and IWISE, local decision-makers can assess which components of water security are most feasible and impactful to target for improvement in their area. In institutional or public settings, measures of water source or reliability of water supply may be appropriate alternative measures. Recommendations on these components related to water quantity can be especially useful for planning water supplies in public and institutional settings that serve large populations.

Given these complexities and the lack of empirical data currently available, further research is critical to establish minimum water requirements that support hand hygiene both independently and within the context of broader domestic, public and institutional needs.

### Quantity of soap

We found a lack of observational data on the quantities of soap available to users in community settings. The presence or absence of soap was reported in many studies, though this alone was not enough to determine the quantity of soap present or needed for sustained handwashing. Two observational studies reported on the quantity of soap distributed in refugee camp settings, though they did not explore the relationship between soap quantity present in households and hand hygiene practices.^35 36^ Although not intervention studies, these studies also reflect a unique context in which hygiene materials are regularly distributed to individuals in humanitarian settings. Interestingly, one included study assessed the reported priority use of the distributed soap and determined that laundry was the highest priority, followed by bathing, then either dishes or handwashing in all three countries (Thailand, Kenya and Ethiopia),^35^ aligning with other literature on the prioritisation of soap in domestic settings, including one study in Uganda that reported the same priority order.^20^,^23^ As with water, the domestic setting presents multiple competing needs for soap that must be taken into account when determining recommendations for the quantity needed.^23 35^ Further research is needed to determine how much soap is necessary for effective and sustained handwashing in diverse settings, particularly in low-resource environments. This question of how much soap is needed to enable sustained handwashing in community settings may be more clearly examined in intervention studies with prescribed soap distribution or in an observational study primarily dedicated to this research question.

### Location of hand hygiene facility

Evidence from the literature indicates that the presence of a designated facility in any location supports the practice of hand hygiene; however, the data were insufficient to generate consensus or provide more detail on where facilities should be located. The included studies were primarily conducted in domestic and school settings rather than public settings. Hand hygiene facilities were most frequently assessed ‘in or near latrine/toilet facilities’ (76% of studies in domestic settings and 42% of studies in institutional settings). Most studies that examined the association between location of handwashing facilities and hand hygiene practices focused on the presence or absence of a facility in key locations, often broadly defined. Of these, the majority found a positive association between hand hygiene practices and the presence of a hand hygiene facility in any location, when compared with not having a facility at all. This finding is supported by a meta-analysis from national surveys that found hand washing with soap was about two times more likely to occur when there was a designated facility present compared with no designated facility present.^76^

The aforementioned companion review by our team also identified ‘hand hygiene facilities location’ as a barrier/enabler to hand hygiene across domestic, institutional and public settings—noting that inconvenient location of handwashing facilities limited hand hygiene practice, and the inverse enabled practice.^24^ It is well established that hand hygiene practices decrease as distance between a handwashing facility and the home or latrine increases.^77^ This suggests that, while simply having a designated facility available promotes handwashing, there are certain locations that are more desirable than others for handwashing facilities. These locations are likely those in proximity to key hand hygiene moments (eg, before meals, after using the toilet or entering a public space) in specific locations (eg, near food preparation areas, bathrooms or entrances). However, given the number of potentially different locations that require hand hygiene in key moments in a singular domestic, institutional or public setting, further studies are needed to determine which specific locations of hand hygiene facilities have more significant effects on hand hygiene practices, particularly in low-resource settings where the number of facilities built and supplied may be limited.

### Number of hand hygiene facilities per area or user

When it comes to the number of hand hygiene facilities required for sustained handwashing, it is unclear from the available data whether having multiple facilities has a significant effect on hand hygiene practices or how many may be required per user. We found few studies reporting on the number of hand hygiene facilities per area or user; of a total of four studies, only two reported associations with hand hygiene practices and each was in different settings and with mixed results. One study reported that the presence of multiple sinks in a restaurant area was associated with workers practising ‘appropriate’ handwashing (defined above),^47^ while another study in a daycare centre setting found that multiple sinks per caregiver had no association with handwashing.^67^ Though these measures are not identical, they can be interpreted as measures of accessibility of hand hygiene facilities to workers. No conclusions can be drawn from this small sample of data. Zomer *et al*’s study in the daycare centre, though finding null associations between the number of sinks per caregiver and soap facilities per caregiver, found a significant association between the number of towel facilities per caregiver and handwashing practices.^67^ This study provides a good model of considering the different components of hand hygiene materials separately for their individual effects on hand hygiene that future studies should employ.

### Design of hand hygiene facility

We sought to understand primary considerations for ensuring equitable access to minimum requirements to hand hygiene, yet we found no literature that looked at material availability or its relationships with hand hygiene through an equity lens. Further research on equity is warranted given that a lack of access is known to disproportionately affect certain groups, including women and girls and people with disabilities.^9 78^ We must note that thorough determination of considerations for equitable access to minimum requirements will vary greatly throughout different environmental, sociocultural and economic contexts. We did identify two studies that considered the design of hand hygiene facilities that may impact accessibility and usability. These found that having a permanent or fixed hand hygiene facility was beneficial for hand hygiene practices compared with a mobile facility, suggesting that the specifics of hand hygiene facility design are important to consider. Therefore, recommendations on how to assess individual contexts to determine what would ensure equitable access to these requirements may be most useful and should be published to improve this body of literature.

### Strengths and limitations

This review was part of an integrated protocol for multiple related reviews, which included an exhaustive search strategy encompassing over 20 000 records of peer-reviewed and grey literature that were identified via multiple databases, searching trial registries, handsearching reference lists of systematic reviews, and engaging experts for work yet to be published to identify relevant literature of hand hygiene in community settings. To our knowledge, this is the first review to systematically search the literature for observational studies addressing the minimum requirements for effective hand hygiene in community settings. This review may be critiqued for not using the ‘pearl growing’ technique—searching reference lists of included studies—to identify other articles for potential inclusion. While such an approach could identify additional studies, the approach is time and resource intensive without any guarantee of additional yield. Most importantly, we decided not to add this approach (and adhere to the protocol as published) because we are confident that the robustness of our search process is one of the strengths of this review. Owing to the comprehensive nature of our search, this review includes studies from diverse global contexts from four of six WHO regions and across all three community setting types (domestic, institutional and public).

Despite these strengths, restrictions and limitations should be acknowledged. Most significantly, because there is insufficient data in the literature regarding material requirements for hand hygiene specific to our research questions, we were unable to perform planned meta-analyses or establish a consensus across different contexts and hand hygiene outcomes for our research questions. Our research questions were focused on observational data in natural community settings, and many required studies to report on quantities and spacing, not just presence and absence. This scope was intentionally designed to inform the development of international guidelines that require this information but may have excluded closely related studies that examined hand hygiene materials more broadly or reported related information that is not quantitative. A study with a wider scope that includes experimental data would likely identify additional studies; we did not do this because our intention was to gather data on natural community settings and not intervention contexts. Future reviews could also benefit from including studies that report on alternative or proxy measures for water quantity such as measures of water availability, proximity, reliability, insecurity and source to assess a more comprehensive view of water access. We hope that more studies use these proxy measures in the future to enable this kind of review. This review was also limited by only including articles published in English, which may have excluded relevant data published in other languages and restricted this review’s geographical representation. Despite—and due to—the lack of data and the stated limitations, the review has highlighted key gaps in existing research that are key to determining the needs for minimum requirements for hand hygiene.

Another consideration when interpreting the results presented in this review is the potential for publication bias. We found that material requirements for handwashing were rarely the primary focus of studies, so the reporting of associations within a paper may have been selective towards statistically significant results. Future research should commit to reporting all measures as planned and not avoid publishing results that are not significant or show no effect; publication of such findings is critical for enabling balanced reviews. We also note the difficulty in assessing the potential influence of contextual factors such as cultural norms and other external factors, such as the COVID-19 pandemic, as confounders or moderators of the relationship between material requirements and handwashing practices. For example, the response to the COVID-19 pandemic in many areas focused both on increasing the availability of handwashing materials and messaging promoting hand hygiene behaviours. However, areas that were not able to improve infrastructure and material availability may have still seen increased rates of hand hygiene due to the increased hand hygiene promotion and perceived need. Though we extracted data on these relevant contextual factors, including COVID-19, the available data were not sufficient to provide evidence on the influence of contextual factors on reported relationships of minimum material requirements with hand hygiene. This further emphasises the need for dedicated research on these relationships.

## Conclusions

This systematic review highlights key gaps in our understanding of the minimum requirements necessary for effective and sustained hand hygiene in community settings. While many recommendations exist on specific quantities of water and soap required, as well as the ideal locations, number of users and design considerations for hand hygiene facilities, these recommendations are generally based on expert opinion and estimates rather than robust evidence. There is a clear need for evidence to guide these recommendations in the future to enhance the progress of the hand hygiene sector and improve hand hygiene practices globally. Field research can be costly and challenging, and so opportunities to collect and report this kind of data within funded projects should be considered. Future research focusing on hand hygiene practice should seek to integrate observations about materials requirements for handwashing, quantifying and describing these materials and (where possible) relating them to the hand hygiene outcomes of interest in these studies.

## Supplementary material

10.1136/bmjgh-2025-018926 online supplemental file 1

10.1136/bmjgh-2025-018926 online supplemental file 2

10.1136/bmjgh-2025-018926 online supplemental file 3

10.1136/bmjgh-2025-018926 online supplemental file 4

10.1136/bmjgh-2025-018926 online supplemental file 5

10.1136/bmjgh-2025-018926 online supplemental file 6

10.1136/bmjgh-2025-018926 online supplemental file 7

10.1136/bmjgh-2025-018926 online supplemental file 8

10.1136/bmjgh-2025-018926 online supplemental file 9

## Data Availability

Data are available in a public, open access repository.
