# Supplementary material for: Minimum material requirements for hand hygiene in community settings: a systematic review
Source: BMJ Glob Health. 2025 Sep 16;10(Suppl 7):e018926. doi: 10.1136/bmjgh-2025-018926 (PMC12443185; doi:10.1136/bmjgh-2025-018926)
Supplement: online supplemental file 1 [file bmjgh-10-Suppl_7-s001.docx]

**S1** – Research question and eligibility criteria in SPIDER format

| **Research question** | | **Sample** | **Phenomenon of interest** | **Design** | **Evaluation** | **Research type** |
| --- | --- | --- | --- | --- | --- | --- |
| RQ2. What are the minimum requirements (material needs) for the sustained practice of effective hand hygiene in community settings? | (2a) What quantity of water is required to enable handwashing with soap and water at key moments? | General population in community settings | Quantity of water required for handwashing with soap at key moments both as recommended and as commonly practiced | Observational study | Hand hygiene practice (i.e., any action of hand cleansing for the purpose of removing or deactivating pathogens from hands) | Quantitative, mixed methods |
|  | (2b) What quantity of soap is required to enable handwashing with soap and water at key moments? | General population in community settings | Quantity of soap required for handwashing with soap at key moments both as recommended and as commonly practiced | Observational study | Hand hygiene practice (i.e., any action of hand cleansing for the purpose of removing or deactivating pathogens from hands) | Quantitative, mixed methods |
|  | (2c) Where should soap and water or alternatives be located in community settings to enable hand hygiene at key moments? | General population in community settings | Location of soap and water required for handwashing with soap at key moments | Observational study | Hand hygiene practice (i.e., any action of hand cleansing for the purpose of removing or deactivating pathogens from hands) | Qualitative, quantitative, mixed methods |
|  | (2d) What is the optimal spacing and number of users per hand hygiene facility in household settings and public places to enable hand hygiene with soap and water at key moments? | General population in community settings | Spacing and number of users per hand hygiene facility required for handwashing with soap at key moments | Observational study | Hand hygiene practice (i.e., any action of hand cleansing for the purpose of removing or deactivating pathogens from hands) | Qualitative, quantitative, mixed methods |
|  | (2e) What are the main considerations for ensuring equitable access to minimum material requirements and preventing discrimination in community settings? | General population in community settings | Considerations (including location and design) leading to harm or inequitable access to handwashing with soap at key moments or discrimination | Observational study | Hand hygiene practice (i.e., any action of hand cleansing for the purpose of removing or deactivating pathogens from hands) | Qualitative, quantitative, mixed methods |
