# Supplementary figures and images for: Minimum material requirements for hand hygiene in community settings: a systematic review
Source: BMJ Glob Health. 2025 Sep 16;10(Suppl 7):e018926. doi: 10.1136/bmjgh-2025-018926 (PMC12443185; doi:10.1136/bmjgh-2025-018926)

**S3 -** PRISMA flow diagram.


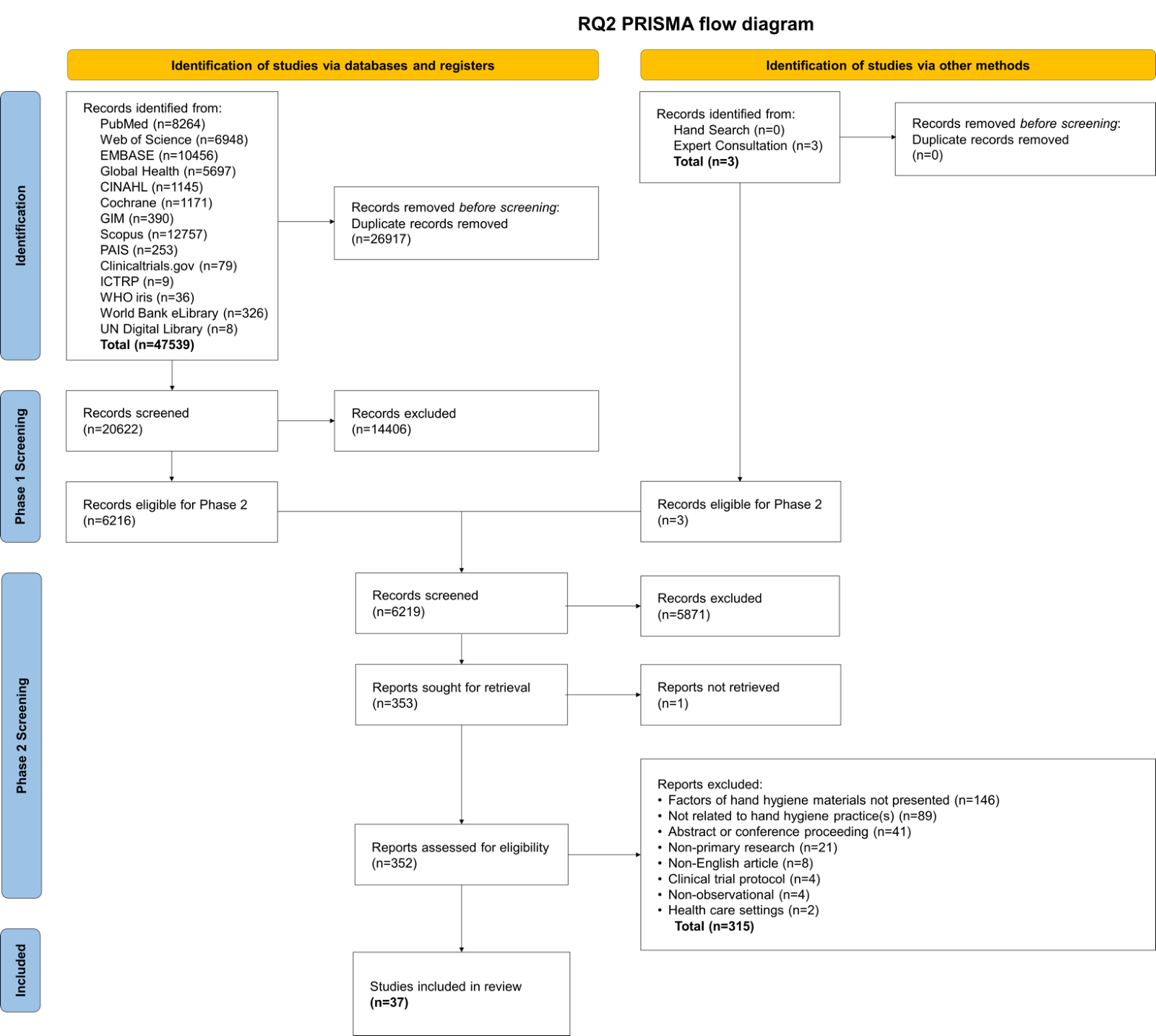

Supplement: online supplemental file 3 [file bmjgh-10-Suppl_7-s003.docx]
