# Supplementary material for: Minimum material requirements for hand hygiene in community settings: a systematic review
Source: BMJ Glob Health. 2025 Sep 16;10(Suppl 7):e018926. doi: 10.1136/bmjgh-2025-018926 (PMC12443185; doi:10.1136/bmjgh-2025-018926)
Supplement: online supplemental file 4 [file bmjgh-10-Suppl_7-s004.docx]

**S4 –** Covidence extraction form template

| **#** | **Field** | **Details** | **Entry** | **Source** |
| --- | --- | --- | --- | --- |
| **1. Information about the study** | | | | |
| 1.1 | Publication type |  | *Select one*  (1) Journal article  (2) Grey literature (e.g., unpublished academic papers [e.g., thesis], non-peer reviewed papers, research and committee reports, government reports, conference papers/abstract, ongoing research)  (777) Other - specify |  |
| 1.2 | Study design |  | *Select one*  (1) Descriptive (survey)  (2) Descriptive (qualitative)  (3) Descriptive (mixed methods)  (4) Randomized controlled trial  (5) Non-randomized control trial  (6) Quasi-experimental  (7) Case-control  (8) Cross-sectional  (9) Cohort  (10) Non-primary research  (777) Other – specify |  |
| 1.3 | Registered trial | Does the study report that it is linked to a registered trial? (e.g., clinicaltrials.gov, ICTRP) | *Select one*  (1) Yes  (0) No |  |
| 1.4 | Trial # | If linked to a registered trial, paste trial registration number | Text  (999) Not applicable |  |
| **2. Eligibility** | | | | |
| 2.1 | Confirm participants/sample | Does the study include general populations in community settings?  DO NOT PROCEED IF “NO” | *Select one*  (1) Yes  (0) No | Phase 2 criteria |
| 2.2 | Confirm phenomena of interest | Check each of the following phenomena of interests that the study includes.  DO NOT PROCEED IF NONE ARE CHECKED | *Select multiple*  (1) Quantity of water required for handwashing with soap at key moments both as recommended and as commonly practiced  (2) Quantity of soap required for handwashing with soap at key moments both as recommended and as commonly practiced  (3) Location of soap and water required for handwashing with soap at key moments  (4) Spacing and number of users per hand hygiene facility required for handwashing with soap at key moments  (5) Considerations (including location and design) leading to harm or inequitable access to handwashing with soap at key moments or discrimination |  |
| 2.3 | Confirm study design | Is the design of the study an observational study?  DO NOT PROCEED IF “NO” | *Select one*  (1) Yes  (0) No |  |
| 2.4 | Confirm evaluation | Does the study include the evaluation of hand hygiene practice (i.e., any action of hand cleansing for the purpose of removing or deactivating pathogens from hands)?  DO NOT PROCEED IF “NO” | *Select one*  (1) Yes  (0) No |  |
| 2.5 | Confirm research type | What research type does the study use?  DO NOT PROCEED IF NONE ARE CHECKED | *Select one*  (1) Qualitative  (2) Quantitative  (3) Mixed methods |  |
| **3. Setting** | | | | |
| 3.1 | Country | Which country is represented in the study? (List all countries separated by a comma, if study is from multiple sites) | Text  999 = Not applicable |  |
| 3.2 | Region | Which region is represented in the study? Which region is represented in the study? | *Check multiple*  (1) Africa  (2) Asia  (3) Europe  (4) Latin America/Caribbean  (5) Middle East  (6) North America  (8) Oceania  (10) Unspecified  (999) Not applicable | WHO reporting requirement |
| 3.3 | Urban/Rural | Does the setting of the population fall under any of these specific categories? Select all that apply | *Check multiple*  (1) Urban  (2) Rural  (3) Peri-urban  (777) Other - specify  (888) Not reported |  |
| 3.4 | Community setting | Does the setting of the population fall under any of these specific categories? Select all that apply | *Check multiple*  (1) Domestic - Households  (2) Public - Markets  (3) Public - Public transportation hubs  (4) Public - Parks, squares, or other public outdoor spaces,  (5) Institutions - Workplace  (6) Institutions - Schools  (7) Institutions - Universities  (8) Institutions - Places of worship  (9) Institutions - Prisons and places of detention  (10) Internally displaced people camps  (777) Other - specify  (888) Not reported | [Macleod et al](https://bmjopen.bmj.com/content/13/6/e068887) |
| **4. Methods** | | | | |
| 4.1 | Aim of study | Paste the aim/objective/purpose/goal as stated in the full text article for the study | Text  (888) Not reported |  |
| 4.2 | Primary study outcome | What was the primary outcome for this study?  Select all that apply | *Select one*  (1) Hand hygiene  (2) Diarrheal diseases  (3) Respiratory infections  (4) Influenza  (5) Infectious diseases  (6) Nutrition  (7) Mental/social well being  (8) Neglected tropical diseases  (9) School absenteeism  (10) COVID-19  (11) Food hygiene  (777) Other – specify(999) Not applicable |  |
| 4.3 | Start date | What is the study start date? Month, Year | Text  (888) Not reported |  |
| 4.4 | End date | What is the study end date? Month, Year | Text  (888) Not reported |  |
| **5. Participants** | | | | |
| 5.1 | Study participants | What is the group of people that researchers are examining in the study? Select all that apply | *Check multiple*  (1) General population  (2) Adults (Women and Men)  (3) Adults (Women only)  (4) Adults (Men only)  (5) Children (Girls and Boys)  (6) Children (Girls only)  (7) Children (Boys only)  (8) Mother-child dyads  (9) Food workers  (10) Non-food occupational workers  (777) Other – specify  (888) Not reported |  |
| 5.2 | Vulnerable populations | Does study concern any of the following vulnerable populations?  Select all that apply | *Check multiple*  (1) Individuals with specific illness or risk factors  (2) Specific ethnic or religious groups  (3) Persons experiencing homelessness  (4) Persons with disabilities  (5) Immigrants and migrants  (6) Refugees and displaced persons (7) Elderly  (8) Pregnant women  (777) Other – specify  (999) None of the above |  |
| 5.3 | Number of participants | What is the total number of participants/sample size?    If the study does not stratify participants by sex or gender, indicate N/A in the Female and Male fields | Table  2 x 3 table for Adult/Child vs Female/Male/Total |  |
| 5.4 | Age group | What is the age range of study participants?  888 = not reported  999 = not applicable | Table  2x2 table for Adult/child vs Lower age/Upper age |  |

# Bias assessment

| **Bias Assessment** | | | | |
| --- | --- | --- | --- | --- |
| MMAT (All articles) | | | | |
| S1 | Screening question 1 (for all types) | Are there clear research questions? | (0) No  (1) Yes  (999) Can’t tell | [MMAT User Guide](http://mixedmethodsappraisaltoolpublic.pbworks.com/w/file/fetch/127916259/MMAT_2018_criteria-manual_2018-08-01_ENG.pdf) |
| S2 | Screening question 2 (for all types) | Do the collected data allow to address the research questions? | (0) No  (1) Yes  (999) Can’t tell | [MMAT User Guide](http://mixedmethodsappraisaltoolpublic.pbworks.com/w/file/fetch/127916259/MMAT_2018_criteria-manual_2018-08-01_ENG.pdf) |
| Qualitative | | | | |
| 1.1 | Is the qualitative approach appropriate to answer the research question? | | (0) No  (1) Yes  (999) Can’t tell | [MMAT User Guide](http://mixedmethodsappraisaltoolpublic.pbworks.com/w/file/fetch/127916259/MMAT_2018_criteria-manual_2018-08-01_ENG.pdf) |
| 1.2 | Are the qualitative data collection methods adequate to address the research question? | | (0) No  (1) Yes  (999) Can’t tell | [MMAT User Guide](http://mixedmethodsappraisaltoolpublic.pbworks.com/w/file/fetch/127916259/MMAT_2018_criteria-manual_2018-08-01_ENG.pdf) |
| 1.3 | Are the findings adequately derived from the data? | | (0) No  (1) Yes  (999) Can’t tell | [MMAT User Guide](http://mixedmethodsappraisaltoolpublic.pbworks.com/w/file/fetch/127916259/MMAT_2018_criteria-manual_2018-08-01_ENG.pdf) |
| 1.4 | Is the interpretation of results sufficiently substantiated by data? | | (0) No  (1) Yes  (999) Can’t tell | [MMAT User Guide](http://mixedmethodsappraisaltoolpublic.pbworks.com/w/file/fetch/127916259/MMAT_2018_criteria-manual_2018-08-01_ENG.pdf) |
| 1.5 | Is there coherence between qualitative data sources, collection, analysis and interpretation? | | (0) No  (1) Yes  (999) Can’t tell | [MMAT User Guide](http://mixedmethodsappraisaltoolpublic.pbworks.com/w/file/fetch/127916259/MMAT_2018_criteria-manual_2018-08-01_ENG.pdf) |
| Quantitative randomized controlled trials | | | | |
| 2.1 | Is randomization appropriately performed? | | (0) No  (1) Yes  (999) Can’t tell | [MMAT User Guide](http://mixedmethodsappraisaltoolpublic.pbworks.com/w/file/fetch/127916259/MMAT_2018_criteria-manual_2018-08-01_ENG.pdf) |
| 2.2 | Are the groups comparable at baseline? | | (0) No  (1) Yes  (999) Can’t tell | [MMAT User Guide](http://mixedmethodsappraisaltoolpublic.pbworks.com/w/file/fetch/127916259/MMAT_2018_criteria-manual_2018-08-01_ENG.pdf) |
| 2.3 | Are there complete outcome data? | | (0) No  (1) Yes  (999) Can’t tell | [MMAT User Guide](http://mixedmethodsappraisaltoolpublic.pbworks.com/w/file/fetch/127916259/MMAT_2018_criteria-manual_2018-08-01_ENG.pdf) |
| 2.4 | Are outcome assessors blinded to the intervention provided? | | (0) No  (1) Yes  (999) Can’t tell | [MMAT User Guide](http://mixedmethodsappraisaltoolpublic.pbworks.com/w/file/fetch/127916259/MMAT_2018_criteria-manual_2018-08-01_ENG.pdf) |
| 2.5 | Did the participants adhere to the assigned intervention? | | (0) No  (1) Yes  (999) Can’t tell | [MMAT User Guide](http://mixedmethodsappraisaltoolpublic.pbworks.com/w/file/fetch/127916259/MMAT_2018_criteria-manual_2018-08-01_ENG.pdf) |
| Quantitative non-randomized | | | | |
| 3.1 | Are the participants representative of the target population? | | (0) No  (1) Yes  (999) Can’t tell | [MMAT User Guide](http://mixedmethodsappraisaltoolpublic.pbworks.com/w/file/fetch/127916259/MMAT_2018_criteria-manual_2018-08-01_ENG.pdf) |
| 3.2 | Are measurements appropriate regarding both the outcome and intervention (or exposure)? | | (0) No  (1) Yes  (999) Can’t tell | [MMAT User Guide](http://mixedmethodsappraisaltoolpublic.pbworks.com/w/file/fetch/127916259/MMAT_2018_criteria-manual_2018-08-01_ENG.pdf) |
| 3.3 | Are there complete outcome data? | | (0) No  (1) Yes  (999) Can’t tell | [MMAT User Guide](http://mixedmethodsappraisaltoolpublic.pbworks.com/w/file/fetch/127916259/MMAT_2018_criteria-manual_2018-08-01_ENG.pdf) |
| 3.4 | Are the confounders accounted for in the design and analysis? | | (0) No  (1) Yes  (999) Can’t tell | [MMAT User Guide](http://mixedmethodsappraisaltoolpublic.pbworks.com/w/file/fetch/127916259/MMAT_2018_criteria-manual_2018-08-01_ENG.pdf) |
| 3.5 | During the study period, is the intervention administered (or exposure occurred) as intended? | | (0) No  (1) Yes  (999) Can’t tell | [MMAT User Guide](http://mixedmethodsappraisaltoolpublic.pbworks.com/w/file/fetch/127916259/MMAT_2018_criteria-manual_2018-08-01_ENG.pdf) |
| Quantitative descriptive | | | | |
| 4.1 | Is the sampling strategy relevant to address the research question? | | (0) No  (1) Yes  (999) Can’t tell | [MMAT User Guide](http://mixedmethodsappraisaltoolpublic.pbworks.com/w/file/fetch/127916259/MMAT_2018_criteria-manual_2018-08-01_ENG.pdf) |
| 4.2 | Is the sample representative of the target population? | | (0) No  (1) Yes  (999) Can’t tell | [MMAT User Guide](http://mixedmethodsappraisaltoolpublic.pbworks.com/w/file/fetch/127916259/MMAT_2018_criteria-manual_2018-08-01_ENG.pdf) |
| 4.3 | Are the measurements appropriate? | | (0) No  (1) Yes  (999) Can’t tell | [MMAT User Guide](http://mixedmethodsappraisaltoolpublic.pbworks.com/w/file/fetch/127916259/MMAT_2018_criteria-manual_2018-08-01_ENG.pdf) |
| 4.4 | Is the risk of nonresponse bias low? | | (0) No  (1) Yes  (999) Can’t tell | [MMAT User Guide](http://mixedmethodsappraisaltoolpublic.pbworks.com/w/file/fetch/127916259/MMAT_2018_criteria-manual_2018-08-01_ENG.pdf) |
| 4.5 | Is the statistical analysis appropriate to answer the research question? | | (0) No  (1) Yes  (999) Can’t tell | [MMAT User Guide](http://mixedmethodsappraisaltoolpublic.pbworks.com/w/file/fetch/127916259/MMAT_2018_criteria-manual_2018-08-01_ENG.pdf) |
| Mixed methods | | | | |
| 5.1 | Is there an adequate rationale for using a mixed methods design to address the research question? | | (0) No  (1) Yes  (999) Can’t tell | [MMAT User Guide](http://mixedmethodsappraisaltoolpublic.pbworks.com/w/file/fetch/127916259/MMAT_2018_criteria-manual_2018-08-01_ENG.pdf) |
| 5.2 | Are the different components of the study effectively integrated to answer the research question? | | (0) No  (1) Yes  (999) Can’t tell | [MMAT User Guide](http://mixedmethodsappraisaltoolpublic.pbworks.com/w/file/fetch/127916259/MMAT_2018_criteria-manual_2018-08-01_ENG.pdf) |
| 5.3 | Are the outputs of the integration of qualitative and quantitative components adequately interpreted? | | (0) No  (1) Yes  (999) Can’t tell | [MMAT User Guide](http://mixedmethodsappraisaltoolpublic.pbworks.com/w/file/fetch/127916259/MMAT_2018_criteria-manual_2018-08-01_ENG.pdf) |
| 5.4 | Are divergences and inconsistencies between quantitative and qualitative results adequately addressed? | | (0) No  (1) Yes  (999) Can’t tell | [MMAT User Guide](http://mixedmethodsappraisaltoolpublic.pbworks.com/w/file/fetch/127916259/MMAT_2018_criteria-manual_2018-08-01_ENG.pdf) |
| 5.5 | Do the different components of the study adhere to the quality criteria of each tradition of the methods involved? | | (0) No  (1) Yes  (999) Can’t tell | [MMAT User Guide](http://mixedmethodsappraisaltoolpublic.pbworks.com/w/file/fetch/127916259/MMAT_2018_criteria-manual_2018-08-01_ENG.pdf) |
