# Supplementary material for: Minimum material requirements for hand hygiene in community settings: a systematic review
Source: BMJ Glob Health. 2025 Sep 16;10(Suppl 7):e018926. doi: 10.1136/bmjgh-2025-018926 (PMC12443185; doi:10.1136/bmjgh-2025-018926)
Supplement: online supplemental file 6 [file bmjgh-10-Suppl_7-s006.docx]

**S6 –** Quality Appraisal of all Included Articles Using the Mixed Methods Appraisal Tool

| **Study** | **Final Score** | **Quantitative Non-randomized Score** | **Quantitative Descriptive Score** | **Mixed Methods Score** | **Criteria from the Mixed Methods Appraisal Tool^1^** | | | | | | | | | | | | | | | | | | | | | | | | |
| --- | --- | --- | --- | --- | --- | --- | --- | --- | --- | --- | --- | --- | --- | --- | --- | --- | --- | --- | --- | --- | --- | --- | --- | --- | --- | --- | --- | --- | --- |
|  |  |  |  |  | KEY  Individual criteria scores can be either 0 (did not meet criteria) or 1 (met criteria); cells that are shaded in gray indicate that a criterion was not applicable to the study type.  Qualitative and quantitative studies were assessed using the five-criteria questionnaire. Mixed methods studies were assessed using the relevant independent questionnaires for qualitative and quantitative work and a five criteria questionnaire for mixed methods; the lowest of the three scores was used as the quality score. Possible scores are 0–5 across study types (5 is the best).  † Indicates that the MMAT was deemed inappropriate for quality appraisal of the article. | | | | | | | | | | | | | | | | | | | | | | | | |
|  |  |  |  |  | **1.1** | **1.2** | **1.3** | **1.4** | **1.5** | **2.1** | **2.2** | **2.3** | **2.4** | **2.5** | **3.1** | **3.2** | **3.3** | **3.4** | **3.5** | **4.1** | **4.2** | **4.3** | **4.4** | **4.5** | **5.1** | **5.2** | **5.3** | **5.4** | **5.5** |
| Adane 2018 | 3 |  | 3 |  |  |  |  |  |  |  |  |  |  |  | 1 | 0 | 1 | 0 | 1 |  |  |  |  |  |  |  |  |  |  |
| Admasie 2016 | 3 |  |  | 3 |  |  |  |  |  |  |  |  |  |  |  |  |  |  |  |  |  |  |  |  | 1 | 1 | 0 | 0 | 1 |
| Admasie 2022 | 4 |  | 4 |  |  |  |  |  |  |  |  |  |  |  | 1 | 0 | 1 | 1 | 1 |  |  |  |  |  |  |  |  |  |  |
| Agaro 2022 | 5 | 5 |  |  |  |  |  |  |  |  |  |  |  |  |  |  |  |  |  | 1 | 1 | 1 | 1 | 1 |  |  |  |  |  |
| Aithal 2014 | 4 | 4 |  |  |  |  |  |  |  |  |  |  |  |  |  |  |  |  |  | 1 | 1 | 0 | 1 | 1 |  |  |  |  |  |
| Belachew 2018 | 4 |  | 4 |  |  |  |  |  |  |  |  |  |  |  | 1 | 1 | 1 | 0 | 1 |  |  |  |  |  |  |  |  |  |  |
| Berhanu 2022 | 4 |  | 4 |  |  |  |  |  |  |  |  |  |  |  | 1 | 0 | 1 | 1 | 1 |  |  |  |  |  |  |  |  |  |  |
| Besha 2016 | 4 |  | 4 |  |  |  |  |  |  |  |  |  |  |  | 1 | 1 | 1 | 0 | 1 |  |  |  |  |  |  |  |  |  |  |
| Biran 2012 | 5 |  |  | 5 |  |  |  |  |  |  |  |  |  |  |  |  |  |  |  |  |  |  |  |  | 1 | 1 | 1 | 1 | 1 |
| Chidziwisano 2019 | 5 |  |  | 5 |  |  |  |  |  |  |  |  |  |  |  |  |  |  |  |  |  |  |  |  | 1 | 1 | 1 | 1 | 1 |
| Fielmua 2021 | 5 |  |  | 5 |  |  |  |  |  |  |  |  |  |  |  |  |  |  |  |  |  |  |  |  | 1 | 1 | 1 | 1 | 1 |
| George 2022 | 4 |  | 4 |  |  |  |  |  |  |  |  |  |  |  | 1 | 1 | 1 | 0 | 1 |  |  |  |  |  |  |  |  |  |  |
| Green 2007 | 5 |  |  | 5 |  |  |  |  |  |  |  |  |  |  |  |  |  |  |  |  |  |  |  |  | 1 | 1 | 1 | 1 | 1 |
| Jubayer 2022 | 5 |  | 5 |  |  |  |  |  |  |  |  |  |  |  | 1 | 1 | 1 | 1 | 1 |  |  |  |  |  |  |  |  |  |  |
| Kalam 2021 | 5 |  |  | 5 |  |  |  |  |  |  |  |  |  |  |  |  |  |  |  |  |  |  |  |  | 1 | 1 | 1 | 1 | 1 |
| Kalumbi 2020 | 5 |  |  | 5 |  |  |  |  |  |  |  |  |  |  |  |  |  |  |  |  |  |  |  |  | 1 | 1 | 1 | 1 | 1 |
| Kebede 2022 | 5 |  | 5 |  |  |  |  |  |  |  |  |  |  |  | 1 | 1 | 1 | 1 | 1 |  |  |  |  |  |  |  |  |  |  |
| Letuka 2021 | 5 | 5 |  |  |  |  |  |  |  |  |  |  |  |  |  |  |  |  |  | 1 | 1 | 1 | 1 | 1 |  |  |  |  |  |
| Luby 2009 | 5 |  | 5 |  |  |  |  |  |  |  |  |  |  |  | 1 | 1 | 1 | 1 | 1 |  |  |  |  |  |  |  |  |  |  |
| Melaku 2023 | 5 |  |  | 5 |  |  |  |  |  |  |  |  |  |  |  |  |  |  |  |  |  |  |  |  | 1 | 1 | 1 | 1 | 1 |
| Moffa 2021 | 5 | 5 |  |  |  |  |  |  |  |  |  |  |  |  |  |  |  |  |  | 1 | 1 | 1 | 1 | 1 |  |  |  |  |  |
| Mugambe 2021 | 5 | 5 |  |  |  |  |  |  |  |  |  |  |  |  |  |  |  |  |  | 1 | 1 | 1 | 1 | 1 |  |  |  |  |  |
| Mwapasa 2022 | 5 |  |  | 5 |  |  |  |  |  |  |  |  |  |  |  |  |  |  |  |  |  |  |  |  | 1 | 1 | 1 | 1 | 1 |
| Natnael 2022 | 4 |  | 4 |  |  |  |  |  |  |  |  |  |  |  | 1 | 0 | 1 | 1 | 1 |  |  |  |  |  |  |  |  |  |  |
| Peterson 1998 | 4 |  | 4 |  |  |  |  |  |  |  |  |  |  |  | 1 | 1 | 1 | 0 | 1 |  |  |  |  |  |  |  |  |  |  |
| Prevolsek 2021 | 5 | 5 |  |  |  |  |  |  |  |  |  |  |  |  |  |  |  |  |  | 1 | 1 | 1 | 1 | 1 |  |  |  |  |  |
| Rao 2021 | 5 | 5 |  |  |  |  |  |  |  |  |  |  |  |  |  |  |  |  |  | 1 | 1 | 1 | 1 | 1 |  |  |  |  |  |
| Ray 2011 | 5 | 5 |  |  |  |  |  |  |  |  |  |  |  |  |  |  |  |  |  | 1 | 1 | 1 | 1 | 1 |  |  |  |  |  |
| Serra 2015 | 5 | 5 |  |  |  |  |  |  |  |  |  |  |  |  |  |  |  |  |  | 1 | 1 | 1 | 1 | 1 |  |  |  |  |  |
| Shrestha 2021 | 4 |  | 4 |  |  |  |  |  |  |  |  |  |  |  | 1 | 1 | 1 | 0 | 1 |  |  |  |  |  |  |  |  |  |  |
| Sibiya 2013 | 4 |  |  | 4 |  |  |  |  |  |  |  |  |  |  |  |  |  |  |  |  |  |  |  |  | 1 | 1 | 0 | 1 | 1 |
| Sondari 2020 | 3 |  | 3 |  |  |  |  |  |  |  |  |  |  |  | 1 | 0 | 1 | 0 | 1 |  |  |  |  |  |  |  |  |  |  |
| Steiner-Asiedu 2011 | 4 |  |  | 4 |  |  |  |  |  |  |  |  |  |  |  |  |  |  |  |  |  |  |  |  | 1 | 1 | 1 | 0 | 1 |
| Tessema 2021 | 3 |  | 3 |  |  |  |  |  |  |  |  |  |  |  | 1 | 0 | 1 | 0 | 1 |  |  |  |  |  |  |  |  |  |  |
| van Beeck 2016 | 5 | 5 |  |  |  |  |  |  |  |  |  |  |  |  |  |  |  |  |  | 1 | 1 | 1 | 1 | 1 |  |  |  |  |  |
| Wami 2022 | 4 |  |  | 4 |  |  |  |  |  |  |  |  |  |  |  |  |  |  |  |  |  |  |  |  | 1 | 1 | 1 | 1 | 0 |
| Zomer 2013 | 5 | 5 |  |  |  |  |  |  |  |  |  |  |  |  |  |  |  |  |  | 1 | 1 | 1 | 1 | 1 |  |  |  |  |  |

^1^Hong, Q.N., Pluye, P., et al. Mixed Methods Appraisal Tool (MMAT) Version 2018 User Guide. McGill Department of Family Medicine. 2018.

**Criteria from the MMAT:**

1.1 Is the qualitative approach appropriate to answer the research question?

1.2 Are the qualitative data collection methods adequate to address the research question?

1.3 Are the findings adequately derived from the data?

1.4 Is the interpretation of results sufficiently substantiated by data?

1.5 Is there coherence between qualitative data sources, collection, analysis and interpretation?

2.1 Is randomization appropriately performed?

2.2 Are the groups comparable at baseline?

2.3 Are there complete outcome data?

2.4 Are outcome assessors blinded to the intervention provided?

2.5 Did the participants adhere to the assigned intervention?

3.1 Are the participants representative of the target population?

3.2 Are measurements appropriate regarding both the outcome and intervention (or exposure)?

3.3 Are there complete outcome data?

3.4 Are the confounders accounted for in the design and analysis?

3.5 During the study period, is the intervention administered (or exposure occurred) as intended?

4.1 Is the sampling strategy relevant to address the research question?

4.2 Is the sample representative of the target population?

4.3 Are the measurements appropriate?

4.4 Is the risk of nonresponse bias low?

4.5 Is the statistical analysis appropriate to answer the research question?

5.1 Is there an adequate rationale for using a mixed methods design to address the research question?

5.2 Are the different components of the study effectively integrated to answer the research question?

5.3 Are the outputs of the integration of qualitative and quantitative components adequately interpreted?

5.4 Are divergences and inconsistencies between quantitative and qualitative results adequately addressed?

5.5 Do the different components of the study adhere to the quality criteria of each tradition of the methods involved?
