# Supplementary material for: Minimum material requirements for hand hygiene in community settings: a systematic review
Source: BMJ Glob Health. 2025 Sep 16;10(Suppl 7):e018926. doi: 10.1136/bmjgh-2025-018926 (PMC12443185; doi:10.1136/bmjgh-2025-018926)
Supplement: online supplemental file 7 [file bmjgh-10-Suppl_7-s007.docx]

**S7 -** Measured associations of hand hygiene facility location with hand hygiene practices

| **Study ID** | **Setting type** | **Hand hygiene facility location** | **Comparison group** | **What outcome was assessed?** | **Outcome type** | **Unadjusted outcome statistic** | **p-value (unadjusted)** | **Adjusted outcome statistic** | **p-value (adjusted)** | **Study authors’ appraisal of significance** | **MMAT (Avg. of studies: 4.20)** |
| --- | --- | --- | --- | --- | --- | --- | --- | --- | --- | --- | --- |
| Aithal 2014 | Households | Toilet; bathroom; outside |  | Self-report of handwashing with soap and water after using the toilet | Chi-Square test | Not reported | 0.018 | - | - | Significant | 4 |
| Aithal 2014 | Households | Toilet; bathroom; outside |  | Self-report of handwashing with soap and water after changing diapers/cleaning a child's bottom | Chi-Square test | Not reported | 0.002 | - | - | Significant | 4 |
| Berhanu 2022 | Household | Presence of a handwashing facility in a student’s home | No handwashing facility in student’s home | Self-report of handwashing with soap and water or ash at multiple key moments | Odds ratio | 6.32  (4.00, 9.97) | Not reported | 3.62 (1.57, 8.34) | 0.002 | Significant | 4 |
| Tessema 2021 | Households | Handwashing facility available | Handwashing facility not available | Self-report of "Good hygiene practices" at multiple key moments | Prevalence ratio | - | - | 0.98 (0.91, 1.06) | Not reported | Not significant | 3 |
| Luby 2009 | Households | Inside or near the toilet | Outside the yard | Direct observation of handwashing with soap and water after using the toilet | Odds ratio | - | - | 1.3  (0.74, 2.3) | 0.37 | Not significant | 5 |
| Luby 2009 | Households | Inside or near kitchen | Outside the yard | Direct observation of handwashing with soap and water after using the toilet | Odds ratio | - | - | 1.4  (0.60, 3.2) | 0.44 | Not significant | 5 |
| Luby 2009 | Households | Elsewhere in the yard | Outside the yard | Direct observation of handwashing with soap and water after using the toilet | Odds ratio | - | - | 1.2  (0.64, 2.1) | 0.63 | Not significant | 5 |
| Luby 2009 | Households | No specific place | Outside the yard | Direct observation of handwashing with soap and water after using the toilet | Odds ratio | - | - | 0.33 (0.06, 1.7) | 0.19 | Not significant | 5 |
| Chidziwisano 2019 | Households | Handwashing facility present | No handwashing facility present | Self-report of frequency of handwashing with soap at critical times | Not reported (“ANOVA mean comparison analysis”) | - | ≤ 0.05 | - | - | Significant | 5 |
| Kalam 2021 | Households | Handwashing facility within 10 feet of a latrine or toilet | No handwashing facility within 10 feet of a latrine or toilet | Self-report of handwashing with soap and water after using the toilet | Odds ratio | 3.38  (1.29, 8.88) | 0.01 | - | - | Significant | 5 |
| Sondari 2020 | Schools | Facilities | No facilities | Self-report of handwashing with soap and water at multiple key moments | Odds ratio | 1.94  (1.2, 3.14) | 0.005 | 1.9  (1.15, 3.03) | 0.011 | Significant | 3 |
| Admasie 2022 | Schools | In the school compound | No handwashing facility in school | Self-report of "Proper handwashing practice" at multiple key moments | Odds ratio | 3.44  (2.15, 5.50) | Not reported | 3.84 (3.60, 4.07) | Not reported | Significant | 4 |
| Green 2007 | Restaurants | Hand sink in worker's sight | No hand sink in worker's sight | Direct observation of handwashing with soap and water before, during, and after preparing food | Odds ratio | - | - | 1.93 (1.15, 3.23) | <0.01 | Significant | 5 |
| Natnael 2022 | Barbershops and beauty salons | Presence of hand-washing facility that is convenient and user friendly in/near the barbershop/the beauty salon | No hand-washing facility that is convenient and user friendly | Self-report of hand hygiene with soap and water or ABHR at multiple key moments | Odds ratio | 1.12  (0.76, 1.66) | 0.549 | - | - | Not significant | 4 |
| Natnael 2022 | Barbershops and beauty salons | Presence of hand-washing facility with water and soap | No hand-washing facility | Self-report of hand hygiene with soap and water or ABHR at multiple key moments | Odds ratio | 7.18  (4.64, 11.12) | <0.001 | 5.55 (3.28, 9.4) | <0.001 | Significant | 4 |
