# Supplementary material for: Minimum material requirements for hand hygiene in community settings: a systematic review
Source: BMJ Glob Health. 2025 Sep 16;10(Suppl 7):e018926. doi: 10.1136/bmjgh-2025-018926 (PMC12443185; doi:10.1136/bmjgh-2025-018926)
Supplement: online supplemental file 8 [file bmjgh-10-Suppl_7-s008.docx]

**S8 -** Associations of number of facilities per area or user with hand hygiene practices

| **Study ID** | **Setting type** | **Number of facilities per area or user** | **Comparison group** | **What outcome was assessed?** | **Outcome type** | **Unadjusted outcome statistic** | **p-value (unadjusted)** | **Adjusted outcome statistic** | **p-value (adjusted)** | **Study authors’ appraisal of significance** | **MMAT**  **(Avg. of studies: 5)** |
| --- | --- | --- | --- | --- | --- | --- | --- | --- | --- | --- | --- |
| Green 2007 | Restaurant | Multiple hand sinks | 1 or no sinks | Direct observation of handwashing with soap and water before, during, and after preparing food | Odds ratio | - | - | 1.63  (1.07, 2.47) | <0.05 | Significant | 5 |
| Zomer 2013 | Daycare center | Number of sinks per caregiver | Not applicable | Direct observation of handwashing with soap and water followed by hand drying or use of an ABHR at multiple key moments | Odds ratio | 1.05  (0.71, 1.55) | Not reported | - | - | Not significant | 5 |
| Zomer 2013 | Daycare center | Number of towel facilities per caregiver | Not applicable | Direct observation of handwashing with soap and water followed by hand drying or use of an ABHR at multiple key moments | Odds ratio | 1.37  (0.95, 1.97) | <.2 | 2.33  (1.40, 3.88) | <0.01 | Significant | 5 |
| Zomer 2013 | Daycare center | Number of soap facilities per caregiver | Not applicable | Direct observation of handwashing with soap and water followed by hand drying or use of an ABHR at multiple key moments | Odds ratio | 1.03  (0.71, 1.50) | Not reported | - | - | Not significant | 5 |
