# Supplementary material for: Minimum material requirements for hand hygiene in community settings: a systematic review
Source: BMJ Glob Health. 2025 Sep 16;10(Suppl 7):e018926. doi: 10.1136/bmjgh-2025-018926 (PMC12443185; doi:10.1136/bmjgh-2025-018926)
Supplement: online supplemental file 9 [file bmjgh-10-Suppl_7-s009.docx]

**S9 -** Associations of hand hygiene facility design with hand hygiene practices

| **Study ID** | **Setting type** | **Design of facility** | **Comparison group** | **What outcome was assessed?** | **Outcome type** | **Unadjusted outcome statistic** | **p-value (unadjusted)** | **Adjusted outcome statistic** | **p-value (adjusted)** | **Study authors’ appraisal of significance** | **MMAT**  **(Avg. Of studies: 5)** |
| --- | --- | --- | --- | --- | --- | --- | --- | --- | --- | --- | --- |
| Kalam 2021 | Households | Presence of a permanent handwashing facility in the home | No permanent handwashing facility in the home | Self-report of handwashing with soap and water after using the toilet | Odds ratio | 7.00  (2.33, 21.00) | <0.001 | - | - | Significant | 5 |
| Kebede 2022 | Households | Observed, mobile place | Observed, fixed place | Proxy indicator of handwashing with soap and water (no specified moments) | Odds ratio | 0.15  (0.12, 0.18) | Not reported | 0.19  (0.16, 0.23) | Not reported | Significant | 5 |
| Zomer 2013 | Daycare centers | Paper towels | Both paper and fabric towels | Direct observation of handwashing with soap and water followed by hand drying or use of an ABHR at multiple key moments | Odds ratio | 1.66  (1.13, 2.43) | <0.01 | 2.13  (1.32, 3.44) | <0.01 | Significant | 5 |
| Zomer 2013 | Daycare centers | Fabric Towels | Both paper and fabric towels | Direct observation of handwashing with soap and water followed by hand drying or use of an ABHR at multiple key moments | Odds ratio | 1.15  (0.81, 1.64) | Not reported | 1.45  (0.92, 2.28) | Not reported | Not significant | 5 |
| Zomer 2013 | Daycare centers | Paper towels | Only fabric towels | Direct observation of handwashing with soap and water followed by hand drying or use of an ABHR at multiple key moments | Odds ratio | 1.44  (1.03, 2.02) | Not reported | 1.47  (1.00, 2.16) | Not reported | Significant | 5 |
| Zomer 2013 | Daycare centers | Soap pump | Soap dispenser | Direct observation of handwashing with soap and water followed by hand drying or use of an ABHR at multiple key moments | Odds ratio | 1.29  (0.73, 2.28) | Not reported | - | - | Not significant | 5 |
| Zomer 2013 | Daycare centers | Soap pump and soap bar | Soap dispenser | Direct observation of handwashing with soap and water followed by hand drying or use of an ABHR at multiple key moments | Odds ratio | 1.01  (0.09, 11.73) | Not reported | - | - | Not significant | 5 |
| Zomer 2013 | Daycare centers | Soap pump and soap dispenser | Soap dispenser | Direct observation of handwashing with soap and water followed by hand drying or use of an ABHR at multiple key moments | Odds ratio | 1.25  (0.65, 2.39) | Not reported | - | - | Not significant | 5 |
